# Feasibility of wearable activity tracking devices to measure physical activity and sleep change among adolescents with chronic pain—a pilot nonrandomized treatment study

**DOI:** 10.3389/fpain.2023.1325270

**Published:** 2024-01-25

**Authors:** Ashley Junghans-Rutelonis, Leslie Sim, Cynthia Harbeck-Weber, Emily Dresher, Wendy Timm, Karen E. Weiss

**Affiliations:** ^1^ AJR & Co Consulting and Mental Health, St. Paul, MN, United States; ^2^ Department of Psychiatry & Psychology, Mayo Clinic, Mayo Clinic College of Medicine, Rochester, MN, United States; ^3^ Department of Nursing, Mayo Clinic, Rochester, MN, United States; ^4^ Department of Physical Medicine and Rehabilitation, Mayo Clinic, Rochester, MN, United States

**Keywords:** chronic pain, pediatric, exercise, interdisciplinary, treatment, physical activity, adolescent, wearable device

## Abstract

**Purpose:**

Personal informatics devices are being used to measure engagement in health behaviors in adults with chronic pain and may be appropriate for adolescent use. The aim of this study was to evaluate the utilization of a wearable activity tracking device to measure physical activity and sleep among adolescents attending a three-week, intensive interdisciplinary pain treatment (IIPT) program. We also assessed changes in physical activity and sleep from baseline to the treatment phase.

**Methods:**

Participants (57.1% female, average age 15.88, *SD* = 1.27) wore an activity tracking device three weeks prior to starting and during the treatment program.

**Results:**

Of 129 participants contacted, 47 (36.4%) agreed to participate. However, only 30 (64%) complied with the instructions for using the device prior to programming and during program participation. Preliminary analyses comparing averages from 3-weeks pre-treatment to 3-weeks during treatment indicated increases in daily overall activity minutes, daily step counts, and minutes of moderate to vigorous physical activity (by 353%), as well as a corresponding decrease in sedentary minutes. There was more missing data for sleep than anticipated.

**Conclusions:**

Wearable activity tracking devices can be successfully used to measure adolescent physical activity in-person, with more difficulty obtaining this information remotely. Adolescents with chronic pain experience improvements in objective measurements of physical activity over the course of a 3-week IIPT program. Future studies may want to spend more time working with pediatric patients on their understanding of how to use trackers for sleep and physical activity.

## Introduction

Chronic pain affects approximately 30% of children and adolescents, with 5% reporting moderate to severe pain that significantly interferes with their quality of life (1–4). Concerningly, pain frequently interferes with engagement in healthy behaviors such as sleep and regular physical activity, creating disruptions above and beyond difficulties experienced by peers without chronic pain and often continuing into adulthood (2–9). Sleep disruptions can include extremely short sleep duration (31% of individuals with chronic pain sleep less than 6.5 h) which is associated with greater next day pain and reduced utilization of effective coping skills (10). Additionally, because many youth with chronic pain initially report engagement in physical activity is painful or tiring, they subsequently avoid physical activity. Reduction in physical activity not only leads to increased physical debilitation, but as sedentary behavior increases so does the likelihood of reporting pain (11). Of note, youth with chronic pain who engage in higher daily average activity report lower intensity end-of-day pain (12). Consequently, improvement in sleep and physical activity is an essential component of chronic pain treatment.

Given robust associations between improvements in physical activity and sleep and increased functioning, finding reliable and valid ways to measure these improvements can further enhance chronic pain rehabilitation. Subjective self-report questionnaires are often used to assess type, intensity, and frequency of functioning. However, youth subjective reports of activity, especially moderate to vigorous physical activity, and sleep can be inaccurate and inflated (13). In a systematic review, Adamo and colleagues (14) showed 72% of youth significantly over-reported their moderate to vigorous physical activity and this over-reporting tendency can be especially strong among inactive youth (15). Despite the low reliability and problematic self-reporting biases, much chronic pain research relies solely on self-report methodology to track functioning and sleep. Accelerometers and actigraphy can improve reporting of intensity and amount of activity, reduce reporting bias, highlight differences in actual activity vs. perceived activity (16), and can be a cost-efficient, yet valid tool when compared against more expensive devices (e.g., polysomnography) (17) or paper/pencil questionnaires.

Several studies on intensive interdisciplinary pain treatment (IIPT) programs have included objective assessment of physical functioning. These studies showed post-treatment improvements in walking speed (measured by time taken to walk a 10 meter distance); number of sit/stand movements in one minute (18, 19); improvements in strength, agility, and coordination from pre to post-treatment using the Bruininks-Oseretsky Test of Motor Proficiency (BOT-2) (20, 21); as well as improved timed run (22), and balance, dexterity, and exercise tolerance (23, 24). Additional work has utilized actigraphy monitoring of physical activity and sleep among youth with chronic pain conditions outside of IIPTs (25, 26). However, no studies to date have used wearable activity trackers to report on objective physical activity or sleep measurement changes at pre-treatment and during the course of an IIPT (27).

Adults with chronic pain report beliefs that personal informatics systems can aid in sharing data with health providers to improve communication and treatment and can facilitate a deeper understanding of their own pain self-management and functioning (28). Personal informatics technologies can facilitate objective measurement in IIPTs, quick communication between patients and medical teams, and sustainable personal management of physical activity and sleep data. The possibilities for mobile communication of this technology have the potential to increase the quality of data used to examine and improve outcomes of IIPTs. Wearable activity trackers are an example of personal informatics technology that are utilized by the general population to improve health related behaviors, are shown to provide accurate measurements of physical activity, and have been utilized in treatment programs for adults with chronic pain (29, 30). Due to their low cost and ability to view data in real time, these devices are more accessible than actigraphy and have research supporting their reliability and validity in comparison to actigraphy (31). Commercially available activity trackers are small mobile devices worn on the wrist that contain a pedometer and altimeter, as well as actigraphy technology for measuring diurnal and nocturnal movement. These devices are valid and reliable measures of physical activity (despite concerns about the energy expenditure estimates in adults) (32, 33). However, findings are mixed on whether they are reliable measures of sleep, with some studies showing over-estimates of total sleep time and sleep efficacy (34–39). At this time, research examining the reliability of these devices to measure sleep and physical activity with adolescents is limited (40, 41). Due to difficulties collecting reliable and accurate subjective physical activity and sleep data, especially among youth with chronic pain who are at an increased risk for deconditioning and sleep difficulties, utilization of new technologies, such as wearable activity trackers that can provide objective and sustainable data collection, may be a viable way to measure health improvement during IIPTs.

### The present study

The current study focused on the feasibility of using commercially available wearable activity tracking devices to objectively measure sleep and physical activity among youth with chronic pain participating in an IIPT. It was approved by the institutional review board (IRB) where the study was connected. Data was collected from July, 2015 through May, 2016. Data collection occurred over the course of 3-weeks prior to IIPT participation and continued to the 3-week program end. First, we examined recruitment and adherence to use of the device. Second, we conducted preliminary analyses examining changes in physical activity and sleep from 3-weeks prior to IIPT to the 3-weeks attending IIPT. We hypothesized that the majority of adolescents with chronic pain enrolled in an IIPT would wear the device prior to and during participation to facilitate measurement of physical activity and sleep. Additionally, our preliminary hypothesis was that adolescents would show improvements in sleep and physical activity from pre-program to program participation in an IIPT. We anticipated an increase during IIPT participation, however, due to the preliminary nature of this hypothesis, the amount of movement youth with chronic pain were engaged in prior to IIPT and the magnitude of the increase during IIPT was unknown.

## Methods

### Participants

Participants were 47 adolescents, ages 13–18, and their parents who participated in an intensive interdisciplinary pain treatment (IIPT) program. Five participants agreed to begin the study but did not complete it due to losing their device or not attending programming. There was no difference between the study participants vs. non-participants in regards to demographic (e.g., gender, age, ethnicity) or pain characteristics. All participants in the current study were recruited through the IIPT located at an academic medical center in the Midwest United States. Half of the adolescents were from the Midwest (50%), with the South (23%), West (18%), and Northeast (9%) also being represented. Most adolescents were female (57.10%) and Caucasian (90.5%). Please see Table 1 for additional demographic and illness characteristics. Patients were referred to the IIPT by specialists and primary care physicians within and outside of the hospital's health care system. Exclusion criteria included adolescents who were non-English speakers (of which there were none).

**Table 1:** Demographic information based on patient reported history at program admission (*N* = 42).

	Mean (*SD*) or *n* (%)	
	Range	*M* (*SD*)
Age	13–18	15.88 (1.27)
Gender (female %)	57.1% (24)	
Ethnicity—caucasian	90.5% (38)	
Grade	10th grade (mode)	
School Status*—full Time	35.7% (15)	
Part-time	28.6% (12)	
Home schooled	23.8% (10)	
No longer attending	7.1% (3)	
Home tutoring	2.4% (1)	
Completed school/GED	2.4% (1)	
Pain impacts school attendance	97.6% (41)	
Duration of pain (years)	0.3–18	4.20 (4.02)
Admission BMI	17.35–44.38	26.19 (6.84)
Admission BMI percentile	11–99	74.49 (25.86)
Pain disturbs sleep—yes	76.2% (32)	
Average hours of sleep per night at admission	3–12	7.18 (2.26)

BMI, body mass index.

### Treatment description

The IIPT program is a 3-week outpatient group-based treatment program that has been described in detail elsewhere (42–44). The program provides physical therapy, occupational therapy, recreational therapy, and psychological services (primarily cognitive-behavioral therapy and acceptance and commitment therapy) which are primarily delivered in a group format. The primary focus of treatment is improving functioning and returning to age-appropriate activities. Individualized sessions with the aforementioned providers occur as needed. Physical activity occurs in one hour of daily physical therapy and recreational therapy sessions, as well as yoga classes (typically offered two times per week within the context of methods for advanced relaxation), and daily stretching. In addition, patients are encouraged to engage in social activities in the evenings and weekends. Oftentimes, these social activities are physical in nature (e.g., bowling, rock climbing). Physical therapy consists of exercises to increase strength, endurance, flexibility, and cardiovascular health. Interventions provided for sleep include a group focused on sleep hygiene recommendations and individual counseling provided by program nurses and psychologists that focus on normalization of sleep/wake time and cessation of naps.

### Procedures

Eligible participant parents were called by a member of the research team seven weeks prior to their adolescent's IIPT start date. Once written consent from parents and assent from the youth were received, the wearable activity tracking device, and self- and parent-report questionnaires regarding functioning and sleep quality and hygiene were mailed and collected. If questionnaires were not received within two weeks after the initial contact phone call, a reminder call was made. Adolescents were instructed to wear their device daily on their non-dominant wrist and were asked to put it in sleep mode at night and in wake mode each morning. During each of the three weeks prior to program start, a phone call was made to the family to answer any questions and to remind them about tracking. Phone calls were made directly to parents and did not include contact with the adolescents. While the researchers answered any questions about device use brought up on these calls, they were only able to address questions voiced by the parent. Parents were encouraged to check-in with their adolescents about their device use and to bring any use questions to the next weekly call.

Upon arrival at the IIPT, the activity tracking device was synced to a computer in the physical therapy office and data was collected throughout the 3-week program through wireless syncing with the same computer during physical therapy sessions. The adolescent was not provided with their activity tracking device username or password as to not add self-monitoring of physical activity or sleep as a confound to the present study. The same set of questionnaires sent with the activity tracking device were also given to the adolescent and parent at admission and discharge. Questionnaires included the Pediatric Sleep Questionnaire (parent-report), as well as the Functional Disability Inventory, Adolescent Sleep Hygiene Scale, and the Adolescent Sleep Wake Scale (self-reports). Questionnaires were completed at 3-weeks prior to IIPT admission, at IIPT admission, and at the end of the 3 week IIPT. Inclusion of findings from these questionnaires is beyond the scope of this feasibility study.

A one-page report summarizing pre- and post-data was created and reviewed with the family by their program nurse at discharge. The report contained written and graphical information about pre to post-program changes in steps, distance traveled, and moderate to vigorous physical activity minutes. While time in bed and time asleep were initially included in feedback reports, these variables were removed when the data appeared to be of low quality.

### Measurement of physical activity and sleep

Objective measurement of physical activity and sleep was obtained through use of the Fitbit Flex, a personal informatics device (Fitbit Inc., San Francisco, CA). This device provides data on daily steps, moderate to vigorous physical activity minutes, and total sleep time and quality, as well as other caloric and physical functioning information. Algorithms for the Fitbit Flex data were not included in the manual and were not accessible to the researchers. The device is a small device made of elastometer material which is worn on the non-dominant wrist and uses a MEMS 3-axis accelerometer to measure motion patterns in order to determine distance traveled, steps taken, and sleep quality. Devices were set-up and synched with an institutional computer prior to sending to participants and again when the participant started their IIPT. Set-up information and access to the personalized website (“Fitbit Dashboard”) was not provided to parents or to participants. However, families were sent the Fitbit Flex, charger, wristband, and instructions by mail. Written instructions highlighted important components of the Fitbit Flex use and included details about the following: putting on the Fitbit Flex and adjusting the wristband, use in water conditions, charging, and sleep mode. Participants were given step by step detailed instructions on how to put their device in sleep mode and how to exit sleep mode.

During IIPT participation, data was uploaded to the Fitbit Dashboard when the sensor was within range of the base computer. The website includes the following daily data: minutes of sedentary, light, fairly active, and very active activity, steps, distance, minutes asleep, minutes awake, number of awakenings, and time in bed. Data can be viewed online or exported to an excel file for analysis. Activity tracking devices have shown to be a valid and reliable device for measuring physical activity, yet the literature examining the accuracy of sleep data is sparse and mixed (34, 35, 39). Activity and sleep measures were compared from baseline to post-treatment using paired *t*-tests. Analyses were completed using the Statistical Package for Social Sciences (IBM SPSS Statistics 29) (45).

## Results

### Feasibility and recruitment

**Figure 1:**
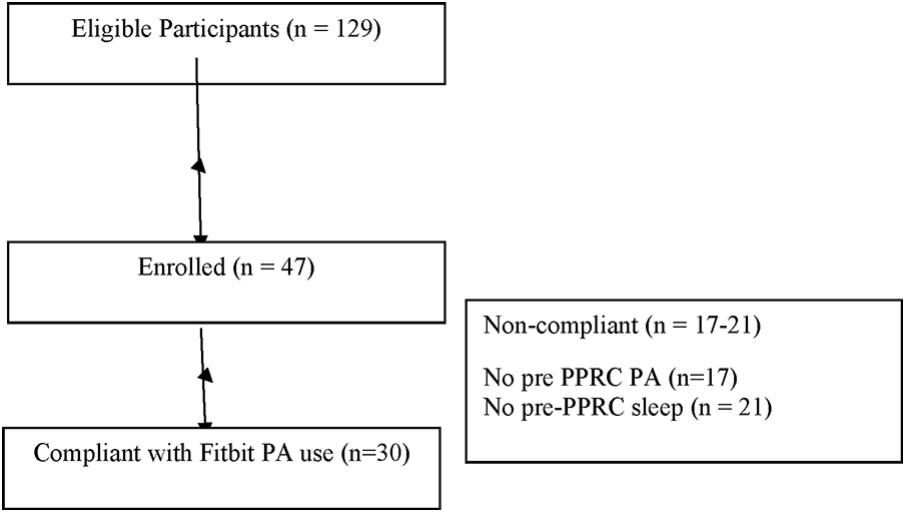
Participant flow chart. PA, physical activity.

One hundred twenty nine adolescents were contacted and 47 (36.4%) eligible participants were recruited to participate in the study. Participation entailed collecting data daily for the 3 weeks prior to IIPT and daily during the 3 weeks of IIPT. Reasons for study non-participation include the following: missed recruitment window due to not responding to study staff phone calls in time (68%), rescheduled date for IIPT and thus missed window (9%), symptom improvement led to withdrawal from IIPT (9%), insurance would not cover IIPT attendance (7%), found device uncomfortable (2%), and other (5%). Of note, 6% of participants who enrolled in the study were sent activity tracking devices but did not participate in the IIPT. The major barrier to recruitment was reaching parents within the time window necessary for study participation, with enough time to send materials if they agreed to participate and to gather 3 weeks of data prior to IIPT. See Figure 1.

### Activity tracking data collection before and during treatment

Of the seventeen (36%) participants who did not record physical activity data and the 21 (45%) participants who did not record sleep data prior to beginning treatment, barriers included “forgetting to wear it, forgetting to put it in sleep mode, and trouble charging.” During the IIPT timeframe, 30 (64%) recorded physical activity and 26 (55%) recorded sleep measurements.

For those who recorded data at both time points (*n* = 30), participants varied in collected days of physical activity data from pre-IIPT (range = 4–22) to during IIPT (range = 4–27). The average number of days of physical activity data significantly increased from 15.67 pre-IIPT (*SD* = 5.38) days to 19.63 (*SD* = 3.55) days during programming; *t* (29) = −4.57, *p* < .001. See Table 2 for additional distribution data.

**Table 2:** Number of days participants collected physical activity and sleep data, reported for those who collected data for both time points.

	Median pre-PPRC	Median during PPRC	Mode pre-PPRC	Mode during PPRC
Physical activity (*n* = 30)	15.5	20	22	20
Sleep (*n* = 26)	4	13	4	9

For those who recorded data at both time points (*n* = 26), participants varied in collected days of sleep from pre-IIPT (range = 1–17) to during IIPT (range 4–23), with an increase in average days collected from 6 (*SD* = 5.09) days pre-IIPT to 13.54 days during IIPT (*SD* = 4.49), *t* (25) = −6.277, *p* < .001).See Table 2 for additional distribution data.

### Objective physical activity and sleep changes before and during treatment

Changes in average steps, miles, minutes sedentary, minutes active, and minutes of moderate to vigorous activity were significant and are outlined in Table 3. Prior to programming, none of the adolescents reported an average of 10,000 steps or more (the World Health Organization recommended average number of daily steps per person) (46). During programming, 40% of adolescents logged an average of 10,000 steps or more per day. Importantly, prior to attending the IIPT, the majority of adolescents (70%) engaged in an average of 5 min or less of moderate to vigorous activity per day (range 0–19). During programming, only 10% of adolescents engaged in 5 min of moderate to vigorous activity or less on average per day. There was a bimodal distribution with the most common average number of minutes being 8 and 14 with an increased range of average minutes of daily moderate to vigorous activity (0–71 min). Two adolescents surpassed the CDC recommended 60 min of moderate to vigorous activity on average per day (47). Overall, moderate to vigorous activity average minutes increased by 353.06% during programming. If the two adolescents who surpassed CDC recommended guidelines are removed as outliers, the change remains significant at an increase of 230.16% during programming.

**Table 3:** Fitbit data collected pre and during PPRC participation (*N* = 30).

	Range	*M* (*SD*)	Range	*M* (*SD*)	*D*	*df*	*t*
Pre-PPRC			During PPRC				
Daily steps	1,123–9,806	5,220.00 (2,146.29)	4,637–20,459	9,320.30 (2,740.32)	1.69	29	9.251***
Daily miles	.06–4.37	2.31 (1.02)	1.94–8.96	4.17 (3.94)	1.79	29	9.808***
Minutes sedentary	754–1,382	1,066.75 (176.75)	608–1,138	827.63 (11.25)	1.36	29	−7.441***
Minutes active	40–330	178.92 (79.39)	33–454	269.28 (74.34)	1.57	29	8.582***
Minutes MVPA	0–19	4.90 (5.32)	0–71	17.30 (14.30)	1.27	29	6.946***

MVPA, moderate to vigorous physical activity.

Averages are presented and change represents change in average steps, miles, and minutes.

Sleep activity, as measured by the wearable tracking device, was unchanged. No change was observed in average minutes of sleep, minutes awake, times awoken during the night, and minutes spent in bed at night. However, pre-IIPT 9 (19.1%) participants were missing sleep data and 12 (25.5%) collected 0 days of sleep data. Given the minimal amount of pre-IIPT sleep data collected, meaningful comparisons with specific sleep changes were not possible.

## Discussion

The current study was conducted to examine the utilization of a commercially available, wearable activity tracking device to ascertain information about physical activity and sleep change among adolescents participating in an IIPT program. The current study is the first to examine use of activity tracking devices among adolescents ages 13–18 and within the context of an IIPT. Prior to program participation, a high percentage of the participants did not use their device on a regular basis, with the modal number of days of use pre-IIPT being zero and during IIPT increasing to nine. However, in the setting of a structured IIPT, adolescents were more adherent to wearing the device for physical activity measurement. Consequently, it appears the device can be utilized for collection of physical activity data among adolescents within the structure of a program setting but utilization was significantly lower when adolescents did not have this structure. Based on the low utilization of the device as directed for sleep, not enough sleep data was collected to allow for examination of objective sleep patterns or changes within an IIPT. Given the challenges found in this study, for adolescents to accurately use this device for collection of sleep data, either a device requiring adolescent engagement to turn on/off sleep mode is not well suited to this age group or, as mentioned previously, future studies should include additional instruction and/or reminders to possibly increase adherence. Findings from this study are consistent with current literature suggesting wearable activity tracking devices can be useful for collecting physical activity data but may be less successful for sleep measurement.

Previous research examining the validity of Fitbit physical activity data collection reported minor concerns regarding under-estimation of energy expenditure, accurate step counts when compared to accelerometer readings, and accurate, slightly over, and slightly under-estimates for distance in studies conducted among healthy adults or those who have a chronic disease or mobility limitations (32, 37, 48). As a whole, the current study suggests utilizing this device to track change in physical activity data in adolescents who have chronic pain, particularly in the context of a structured program, shows promise.

The current study also sought to examine preliminary findings related to change in physical activity prior to IIPT participation compared to activity while participating in the IIPT. Findings from the current study showed significant increases in physical activity for youth with chronic pain during programming compared to their baseline activity level. Given the challenges adolescents had adhering to measurement procedures prior to participation in the program, future efforts to use these devices to obtain activity measurement suggest that adolescents may need more training, regular reminders, or other support to increase adherence.

While evidence shows the majority of adolescents are not meeting recommended levels of physical activity and nearly one quarter are not engaging in any vigorous physical activity at all (47, 49), the findings of this study suggest that adolescents with chronic pain are engaging in even less physical activity than their peers; thus these findings are consistent with the literature (8, 9). In fact, prior to programming, adolescents in the current sample had an average moderate to vigorous activity minutes of zero per day. Importantly, adolescents did not sharply increase from zero to sixty minutes of moderate to vigorous activity during program participation but followed a moderation approach during physical therapy with a goal of continuing to increase moderate to vigorous activity after program discharge. It will be helpful to inform families that participation in an IIPT will assist with increasing activity and this, in turn, can have a positive influence on daily pain intensity and positive affect (12, 50). A program that can provide a structure to increase physical activity can ultimately reduce sedentary behaviors that would otherwise maintain chronic pain including deconditioning, fatigue, and avoidance of activity (11, 25, 51–53). Of note, adolescents engaged in a significant and positive change in activity level over and above what would be expected from a 60-min daily physical therapy session provided in an IIPT or in outpatient treatment. Anecdotally, families reported benefit of seeing this change in activity level when they were provided with reports at the end of programming and felt continuing their physical activities changes post-programming would be feasible and helpful.

Given the significant difficulties with adherence to using the activity tracking device to measure sleep, we cannot draw any conclusions regarding sleep change during the IIPT participation. Adolescents reported difficulty remembering to put the device into sleep mode or use it in general, and others reported some difficulty with charging and “figuring it out,” despite weekly check-in phone calls. Weekly phone calls were directly with parents and specific adolescent concerns may have been missed if parents were unaware of them. Outside of adherence issues, there is a major concern with the high number of night awakenings (24 on average) recorded by the device, as well as the significant missing data from pre- and during program participation. First, the number of awakenings recorded were significantly higher than other published actigraphy-measured awakenings in healthy youth or youth with chronic pain (1.6 and 3.8, respectively) (26). Secondly, Baroni and colleagues expressed similar concerns with missing data, noting up to 70% of college student participants had missing sleep data and 35% had no data at all (54). Similar missing sleep data findings for adults were reported by Ahuja and colleagues (55). If sleep is to be a focal point of any future studies with youth participating in an IIPT, either a significant increase in adherence coaching will need to occur before and during programming or another device with more sensitive sleep informatics would need to be utilized to obtain valid and reliable data.

There are several limitations of this study that should be considered. Due to the homogeneous racial/ethnic composition of the study sample, generalizability is limited. Additionally, from a research perspective, participants were not provided incentives for participation due to limited funding. It is possible incentives may have increased compliance. However, from a clinical feasibility perspective, the finding of this study provides an idea of the utility and limitations of wearable activity tracking devices to measure activity levels and sleep in adolescents with chronic pain. An additional consideration is the lack of randomization in this preliminary study and it is recommended randomization be included in follow-up studies. Furthermore, the particular device used in this study was an early personal informatics model that necessitated the participant select sleep mode. It was selected due to feasibility of cost and it is possible it was less accurate than more expensive and modern options. The cost, however, did make this a feasible option for many families to purchase after program discharge. Future research may benefit from spending more time working with pediatric participants to make sure they understand the details related to sleep mode or other components that necessitate the participant to engage with the device. Text message reminders to continue to engage with the device may also result in increased adherence and improved data collection. Lastly, recruitment was significantly impacted by difficulty reaching participant parents by phone and email. Additional methods of contact, including text messaging, may be helpful to facilitate earlier contact.

## Conclusions

Although research using self-report measures demonstrates that IIPT is effective for youth, limited objective data is available to further support these conclusions. This feasibility study demonstrated that a wearable device appears to be a viable method to measure physical activity before and during an IIPT program, and this physical activity data from wearable devices confirms earlier self-report measures that IIPT helps youth with chronic pain improve their functioning. In addition, families found this objective feedback about physical activity helpful and expressed interest in maintaining use of technology to continue progress at home. Future research should be aimed at collecting clinical informatics on physical functioning after an intensive program and determining how to improve on sleep data collection before, during, and after programming.

## Data Availability

The raw data supporting the conclusions of this article will be made available by the authors, without undue reservation.
